# Dehydroamino acid chemical biology: an example of functional group interconversion on proteins

**DOI:** 10.1039/d0cb00174k

**Published:** 2020-11-06

**Authors:** Lyn H. Jones

**Affiliations:** Center for Protein Degradation, Dana-Farber Cancer Institute 360 Longwood Avenue Boston MA 02215 USA lyn_jones@dfci.harvard.edu

## Abstract

In nature, dehydroalanine (Dha) and dehydrobutyrine (Dhb) residues are byproducts of protein aging, intermediates in the biosynthesis of lanthipeptides and products of bacterial phospholyases that inactivate host kinase immune responses. Recent chemical biology studies have demonstrated the possibility of mapping dehydroamino acids in complex proteomes in an unbiased manner that could further our understanding of the role of Dha and Dhb in biology and disease more broadly. From a synthetic perspective, chemical mutagenesis through site-selective formation of the unsaturated residue and subsequent addition chemistry has yielded homogeneous proteins bearing a variety of post-translational modifications (PTMs) which have assisted fundamental biological research. This Opinion discusses these recent advances and presents new opportunities for protein engineering and drug discovery.

## Abstract 2

The chemical biology of dehydroalanine and dehydrobutyrine in proteins is summarized and new concepts are presented.

## Graphical abstract

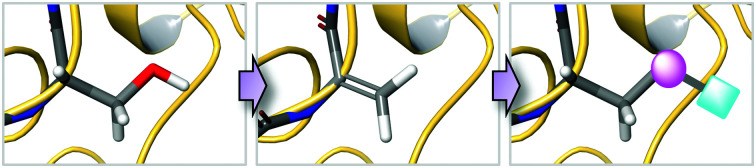

## Dehydroamino acids in nature

**Fig. 1:**
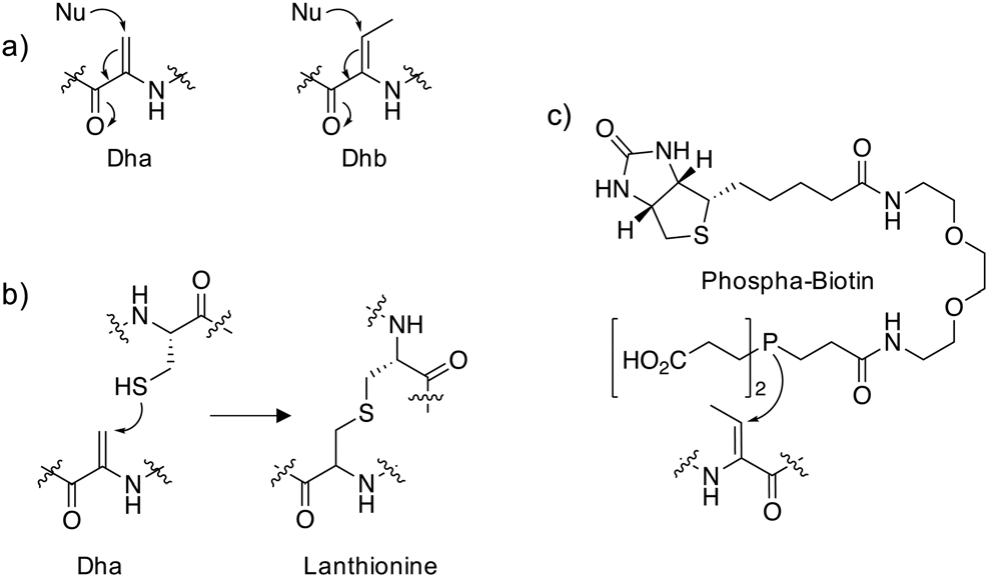
(a) Michael addition chemistry of the unsaturated amino acids dehydroalanine (Dha) and dehydrobutyrine (Dhb). (b) Conjugate addition of cysteine to Dha yields lanthionine. (c) A biotinylated chemical probe for Dhb that utilizes phosphine nucleophilic addition.

Dehydroalanine (Dha) and dehydrobutyrine (Dhb) (Fig. 1a) are naturally occurring amino acids that are formed following dehydration of Ser/Thr or *via* elimination of the phosphorylated residues in peptide natural products and proteins. The following sections describe some important examples that allude to potential further studies in chemical biology.

### Peptides

The thioethers lanthionine and methyllanthionine present in lanthipeptides such as the antibiotic nisin are biosynthesized in a two-step process *via* dehydration of the Ser/Thr residues followed by enzyme-mediated Michael addition of cysteine thiols (Fig. 1b).^1^ Dehydration may proceed through the actions of a dehydratase enzyme or indirectly *via* glutamylation of the Ser/Thr that enhances β-elimination chemistry.^2^ Thioether linkages confer improved metabolic and chemical stability relative to disulfides and restricted conformations of the cyclized peptides often enhance binding affinities by reducing conformational flexibility.^3^ In particular, lanthipeptides are well-suited to the inhibition of protein–protein interactions (PPIs) because they mimic native ligands.^4^ For these reasons, phage display and *in vitro* selection methods have been developed to identify functional lanthipeptides in diverse libraries.^4–6^ Additionally, the promiscuity of lanthipeptide biosynthetic and modification enzymes can be utilized to make novel antimicrobials.^7^ Lysine crosslinking to Dha is also a natural transformation, forming lysinoalanine (Lal) containing lantibiotics such as duramycin and cinnamycin.^8,9^ The preparation of encoded libraries containing lysinoalanine may be feasible using related technologies to those applied to thioether lanthipeptide synthesis which could yield novel lantibiotics with alternative physicochemical properties compared to thioether crosslinked lanthipeptides.

### Proteins

Reports of dehydroalanine formation on proteins are seemingly rare. Pathogenic bacteria deliver effector proteins into host cells to modulate signaling pathways. Bacterial phospholyases such as the *Shigella* type III effector OspF, irreversibly inactivate endogenous mitogen-activated protein kinases (MAPKs) involved in immune signaling through conversion of a phosphothreonine into the Dhb residue which cannot be rephosphorylated.^10^ Using an *in vitro* LC-MS assay that measured the conversion of p-Thr and p-Ser containing peptides to the respective dehydroamino acids, it was possible to assess the substrate specificity of several bacterial phospholyases.^11^ This work suggested that the promiscuous phospholyase activity of OspF may be responsible for the increased pathogenicity of *Shigella* compared to other bacteria such as *Salmonella*.

In order to reveal additional OspF targets, a biotinylated phosphine reagent (Fig. 1c) was developed recently to label Dha and Dhb on proteins that were resistant to Michael addition chemistry using reagents containing thiol and amine nucleophiles.^12^ Importantly, the probe engaged Dhb-containing proteins such as the MAPK ERK1/2 in cell lysate formed by the action of OspF and showed that the histone H3 is also a target of phospholyase activity. In the future, an unbiased mass spectrometry (MS) proteomic analysis of streptavidin-enriched peptides using the biotinylated probe may reveal additional novel dehydroamino acids in proteins. This could help identify novel biomarkers and pathogenic mechanisms following bacterial infection.

Protein aging results in non-enzymatic decomposition to Dha and Dhb, particularly in human lens proteins that are also exposed to the chemical stresses resulting from UV light exposure.^13^ This causes adduct formation with glutathione or protein–protein crosslinking in an age-dependent manner, with higher levels observed in cataract lenses.^14^ Glutathione adducts may prevent crosslinking and inhibit the formation of insoluble aggregates, and in the elderly as glutathione levels decrease in the eye, cataract protein aggregation occurs.

Additional to lantibiotic peptides, the Lal modification is observed in the spirochaete flagella hook protein.^15^ Since the Lal crosslink is responsible for polymerizing the hook subunits, it is required for effective bacterial motility, infection and pathogenicity.^16^ As a result, targeted nucleophilic inhibitors of the protein designed to intercept the Dha intermediate en route to Lal could be developed to treat spirochaetal diseases such as Lyme and leptospirosis. Age-related eliminations to Dha also appear to be responsible for the presence of lysine and histidine crosslinks in cataract lenses, bone, dentin and aorta.^13,17–21^

## Dehydroamino acid synthetic biology

Naturally occurring dehydroamino acids appear to have emerging significance in biology. Additionally, in recent years there have been considerable advances in the synthesis of Dha and Dhb containing proteins for applications in drug discovery and basic research. Several examples are described below that illustrate the breadth of opportunities in the field.

### Phospholyases

**Fig. 2:**
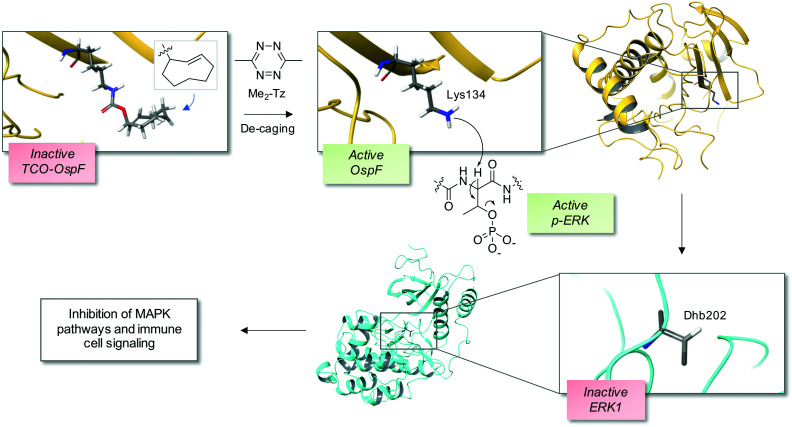
A chemical de-caging strategy enabling control of the phospholyase activity of the bacterial enzyme OspF that irreversibly inactivates ERK1 *via* dehydrobutyrine (Dhb) formation.^23^ A key step in the process is the use of the inverse electron demand Diels–Alder reaction to remove the TCO from the modified OspF to reveal the catalytic lysine that triggers phosphate elimination on p-ERK1 (OspF structure: PDB 3I0U; ERK1 structure: PDB 2ZOQ; TCO = *trans*-cyclooctene; Me_2_-Tz = 3,6-dimethyl-1,2,4,5-tetrazine).

The bacterial phospholyases described above can be directed to inhibit MAPK pathways by targeting them to signaling complexes. OspF was tagged with a leucine zipper and the complementary interacting motif was fused to the scaffold protein in yeast that organizes the osmolarity MAPK pathway, Pbs2, resulting in inhibition of the osmolarity response.^22^ The authors also showed that the phospholyase was able to tune human TCR signaling that could eventually be used to create a safety switch for adoptive immunotherapy. Since the constitutively active OspF irreversibly inhibits MAPK activity, genetically-encoded decaging strategies were recently developed to provide controlled activation of the phospholyase in different contexts, including *in vivo* applications.^23,24^ For instance, a chemically-caged OspF (termed OspF^c^) was engineered to contain a *trans*-cyclooctene (TCO) lysine at the catalytic residue K134 which ablated activity, and an inverse electron demand Diels–Alder reaction with 3,6-dimethyl-1,2,4,5-tetrazine (Me_2_-Tz)^25,26^ released the active parent enzyme (Fig. 2).^23^ Fusion of a nuclear localization sequence to OspF^c^ (to yield nu-OspF^c^) provided a technique for spatio-temporal control of nuclear ERK activity in T cells through the addition of Me_2_-Tz, so avoiding effects on cytosolic feedback regulation. Application of a caged phospholyase in this manner thus afforded another strategy for tuning the T cell response.^23^

### Chemical mutagenesis

**Fig. 3:**
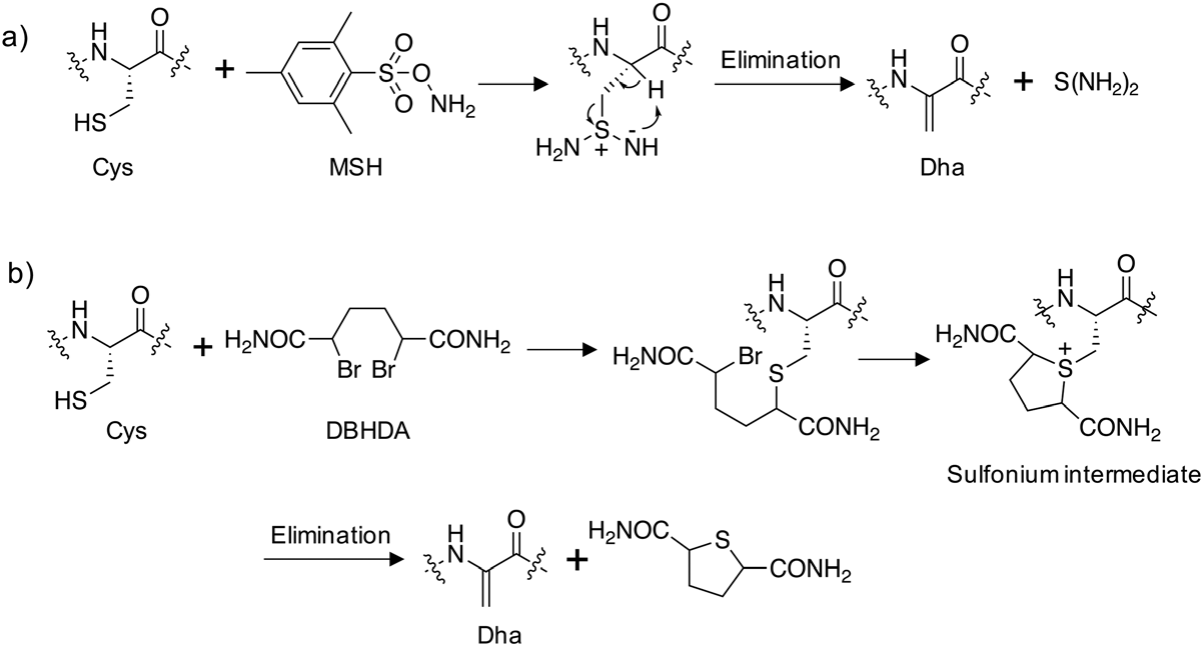
Synthesis of dehydroalanine on proteins from cysteine: (a) amination of cysteine using *O*-mesitylenesulfonylhydroxylamine (MSH) followed by elimination;^33^ (b) alkylation of cysteine using 2,5-dibromohexanediamide (DBHDA) followed by elimination.^34^

Site-directed gene mutagenesis using the 20 standard amino acids and genetic methods of unnatural amino acid incorporation through codon suppression are the main strategies for interrogating and modulating protein function.^27,28^ To significantly expand the diversity of amino acids incorporated into proteins a synthetic methodology termed ‘post-translational chemical mutagenesis’ has been employed to incorporate Dha synthetically into the protein of interest followed by olefin addition chemistry which created a wide-variety of protein modifications.^29,30^ A very early example of Dha formation was reported by the Koshland group where the highly reactive catalytic serine in chymotrypsin was sulfonylated using *p*-toluenesulfonyl chloride and treatment with base resulted in elimination to the Dha derivative yielding an inactive enzyme.^31^ Since then, considerably milder conditions have been developed, particularly by the Davis group, to convert the intrinsically more reactive cysteine amino acid to Dha, enabling subsequent transformations.^32^ Oxidative amidation/Cope-type elimination using *O*-mesitylenesulfonylhydroxylamine (MSH) yielded Dha in subtilisin^33^ and an even milder and more selective reagent was developed, 2,5-dibromohexanediamide (DBHDA), that relies on the bis-alkylation of Cys and subsequent elimination of the sulfonium intermediate (Fig. 3).^34^ DBHDA was used to regioselectively transform single cysteines to Dha residues in bacterial phosphatases.^35^ The study illustrates the utility of Dha as a chemical and spectroscopic reporter for reactivity mapping in proteins containing multiple cysteines.

Deuteration of Dha *via* thia-Michael addition in deuterated media which installs a nonexchangeable backbone deuterium assisted studies of the Dha formation itself.^36^ This work indicated the formation a sulfonium ylide that acts as an internal base and abstracts a proton from the backbone resulting in elimination to give the Dha (analogous to the Swern reaction mechanism).

### PTM mimicry

**Fig. 4:**
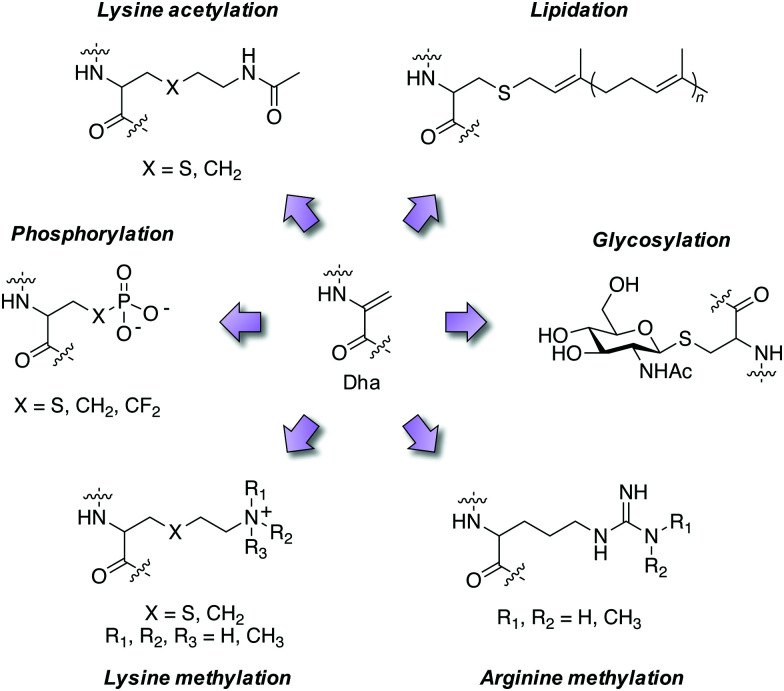
Mimicry of post-translational modifications using dehydroalanine synthetic chemistry.

Employing these reagents and subsequent trapping of the Dha tag using nucleophilic thia-, aza- and selena-Michael additions has delivered diverse site-selective transformations that are mimics of natural PTMs such as phosphorylation, glycosylation, lipidation, lysine methylation and acetylation (Fig. 4).^32,33,37–39^ PTMs are traditionally difficult to obtain in pure form *via* natural sources, but chemical transformations *via* Dha chemistry yield pure modified proteins thus facilitating further research. For example, isolation of homogeneous monophosphorylated proteins is challenging and Asp/Glu point mutations which are often employed for constitutive activation of kinases are poor phosphate mimetics, and this hinders biochemical studies. A single phosphorylation site was incorporated into position 180 of p38α through the reaction of thiophosphate with Dha, and subsequent kinetics studies showed unambiguously that this phosphosite was sufficient to activate the kinase, and that Type II (DFG-out) small molecule binders inhibit phosphorylated p38α.^40^ Several modifications of histone proteins and their study on the influence of chromatin biology have been enabled by Dha chemistry and I point the reader to some excellent reviews of the area.^32,41,42^

### Activity-based probes

Dha has also been incorporated into ubiquitin that allows for covalent adduct formation with the catalytic cysteine in ubiquitin ligases, thus providing an activity-based probe for the enzyme family (*e.g.* labeling of UBE1 in cells was competed by the inhibitor PYR-41).^43^ In a similar manner, Dha probes were developed to study the deubiquitinase enzyme family.^44^ Such technologies advance selectivity determination (particularly when using MS proteomics as a readout) and pharmacokinetic–pharmacodynamic understanding of drug action that enable therapeutic target validation studies using chemical probes through the elucidation of drug–target occupancy.^45^

### Antibody–drug conjugates (ADCs)

Therapeutic utility of Dha conjugation chemistry was demonstrated through the site-selective aza-Michael addition of the anticancer drug crizotinib to Thiomab-Dha targeting the Her2 receptor, resulting in a precise drug-to-antibody (DAR) ratio of 2.^39^ Importantly, the study highlights an advantage of nitrogen nucleophiles compared to commonly used sulfur and phosphorous derivatives by avoiding disulfide cleavage that often compromises the structural and functional integrity of antibodies. Targeted delivery of crizotinib by the ADC enhanced cancer cell killing activity 10-fold. The selectivity and reliability of the chemistry suggests that further expansion of Dha bioconjugation into other therapeutic modalities will emerge in the coming years.

### New chemistry

An important development in Dha synthetic chemistry was reported recently which leveraged radical-based adduct formation to introduce a plethora of diverse modifications.^46,47^ These studies provide the first examples of biocompatible C(sp^3^)–C(sp^3^) formation on proteins and the bioorthogonality of the radical-centered chemistry delivered highly site and regioselective transformations. Another recent study reported the photochemical generation of carbon radicals using visible light that subsequently reacted with Dha residues selectively.^48^ The technique demonstrated broad applicability, with the synthesis of over 50 novel side chains bearing a variety of different functionalities on diverse protein scaffolds.

Genetically encoded fluorosulfate l-tyrosine (FSY) site-selective incorporation and subsequent reaction with proximal serine or threonine residues resulted in proximity mediated sulfur(vi)-fluoride exchange (SuFEx).^49^ Elimination of the crosslinked sulfate diester yielded Dha and Dhb, which enabled proof-of-concept derivatization with thio-saccharide to synthesize a glycoprotein mimetic.

**Fig. 5:**
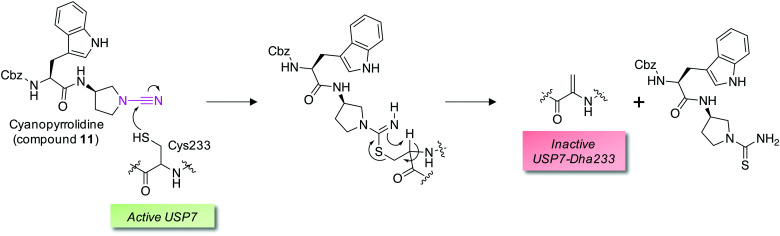
A reversible covalent inhibitor of the deubiquitinase USP7 possessing a cyanamide warhead (magenta, compound **11**) engages the catalytic cysteine and unexpectedly triggers β-elimination to Dha.^52^

**Fig. 6:**
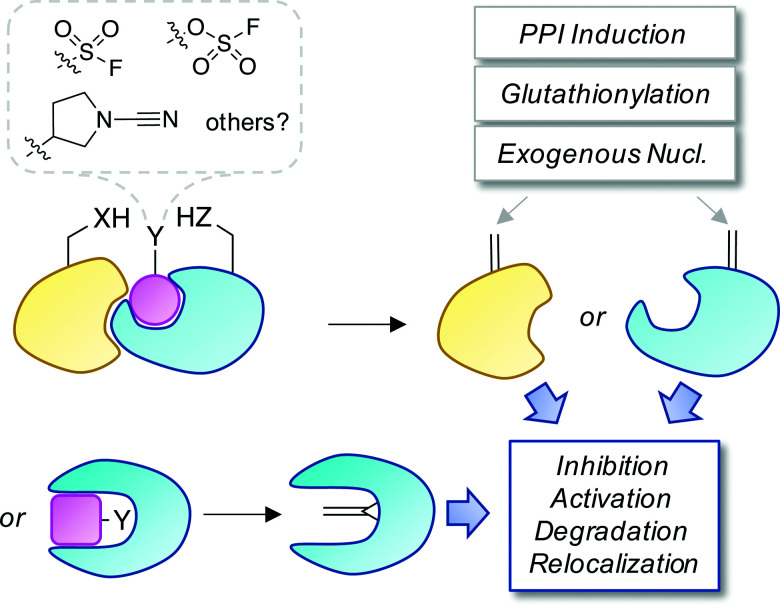
A proposed strategy to chemically control proteins through ligand-mediated functional group interconversion to dehydroamino acids. Activation of Ser/Thr or Cys using sulfur(vi)-fluoride exchange (SuFEx)^56^ or cyanamide chemistry^52^ yielding site-selective Dha or Dhb incorporation (within a binding pocket or on a protein surface) is depicted. The unsaturated amino acids could then be intercepted by neighboring residues, interacting proteins, nucleophilic metabolites such as glutathione, or exogenous nucleophiles causing a loss- or gain-of-function as desired.

Covalent and reversible covalent inhibition of proteins through rational targeting of cysteine residues is a commonly used strategy in medicinal chemistry to enhance potency, pharmacological duration and selectivity. Cyanamides have been used as reversible covalent electrophiles that target reactive cysteine residues in proteins.^50,51^ Recently, a cyanopyrrolidine inhibitor of ubiquitin specific protease 7 (USP7) was developed that labeled the active site cysteine and surprisingly underwent an elimination reaction to create Dha (Fig. 5).^52^ A related enzyme, USP30 was covalently modified by the probe but elimination to Dha did not occur, which appeared to be due to greater flexibility of the USP7 binding site that facilitated α-proton abstraction. These results suggest an intriguing possibility – that small molecule chemical probes could be rationally designed to mediate the site-selective formation of Dha on endogenous proteins in cells and *in vivo*. This could permit chemical control of protein function in novel ways, not just through canonical enzyme inhibition, but also through ‘gain-of-function’ mutagenesis allowing for manipulation of protein location and homeostasis by inducing protein–protein interactions or intercepting the Dha with exogenous nucleophiles (also see Fig. 6).

## Future directions

Although dehydroamino acids are naturally occurring, their study has been limited to the biosynthesis of lanthipeptides, the action of bacterial phospholyases on kinases or as byproducts of protein aging. More research is needed to elucidate the roles of Dha/Dhb in human biology and disease. It will be interesting to know if such modifications result in protein gain- or loss-of-function and whether this leads to therapeutic opportunities. Below are some ideas for further chemical biology research in the area:

• Biotinylated nucleophilic probes could be used in conjunction with unbiased MS analysis to map dehydroamino acids in the proteome and measure changes following bacterial infection or in disease more broadly. Cell permeable clickable functional probes could report on intracellular Dha/Dhb functionality in the more physiologically relevant context of intact cells. This is likely important because the formation of dehydroamino acids is redox sensitive and could thus be compartmentalized within cells, *i.e.* cell lysis may lose biological information.

• The crosslinking of cysteine, lysine and histidine, and potentially other residues, to unsaturated amino acids in peptides and proteins should be further explored. Such chemistry may be an unwanted byproduct that inhibits protein activity, as seen in protein aging, but in certain instances, such as the flagellar hook protein, it may confer an important native function which could be exploited in protein engineering and prospective design.

• The phospholyase activity of bacterial enzymes has been harnessed to control kinases and rewire downstream immune signaling pathways. Further applications of phospholyases in human cells and the development of enzyme delivery vehicles such as lipid nanoparticles, hold considerable promise in synthetic biology with potential *in vivo* applications.^23,24,53^ These enzymes could also be used to advance novel bioconjugation approaches and generate chemically homogeneous biotherapeutics that facilitate rational design and elucidation of structure–function relationships.

• The development of proteolysis-targeting degraders (PROTACs)^54^ and molecular glues^55^ has highlighted prospects for small molecule control of protein homeostasis. Templated or proximity-driven generation of dehydroamino acids using covalent chemical probes, fortuitously demonstrated by the cyanamide USP7 modulator, would appear to be an exciting possibility for the field (one potential strategy is illustrated in Fig. 6). Targeted functionalization and subsequent elimination to Dha/Dhb could not only lead to inhibition of enzymatic activity (as seen for the phospholyase deactivation of MAPKs) but potentially enable further site-selective conjugation in cells. This could include the induction of new protein complexes that bring about protein relocalization or re-wiring of signaling cascades. Electrophile formation can be a toxic event (*e.g.* when glutathione conjugation becomes limiting) and controlled toxic gain-of-function modifications through Dha/Dhb formation could be utilized in therapeutic anticancer strategies.

There appear to be exciting opportunities to develop a toolkit of biochemical transformations and new covalent warhead chemistries to expand the variety of tagging methodologies in cells and *in vivo*. In the coming years, advances in dehydroamino acid chemical biology will likely impact numerous aspects of drug discovery and drive fundamental understanding of biology. More broadly, functional group interconversion of amino acid residues could be a general strategy to control proteins in the future.

## Conflicts of interest

LHJ receives research funding from Deerfield.

## Supplementary Material
